# Author Reply

**DOI:** 10.1111/1471-0528.18013

**Published:** 2024-11-25

**Authors:** Berit Rein Solhaug, Rune Svenningsen, Maria Øyasæter Nyhus, Ingrid Volløyhaug

**Affiliations:** ^1^ Department of Obstetrics and Gynecology St. Olavs Hospital Trondheim Norway; ^2^ Department of Clinical and Molecular Medicine Norwegian University of Technology and Science Trondheim Norway; ^3^ Department of Obstetrics and Gynecology Oslo University Hospital Oslo Norway; ^4^ Institute of Clinical Medicine University of Oslo Oslo Norway

Dear Dr. Papageorghiou,

We would like to extend our gratitude to Rotem and O'Sullivan [1] for their positive and thoughtful comments on our long‐term follow‐up study on mid urethral sling (MUS) surgery. We appreciate their recognition of the importance of performing such long‐term follow‐up studies, especially in surgical methods involving synthetic meshes.

We fully agree on the importance of differentiating between various types of mesh and their distinct risk profiles. We also agree on the important point of careful patient selection and counselling that might increase success rates and minimise complication risks. One excellent example is the association between higher BMI and lower subjective cure rates found in our study and also later confirmed in a recent study from Sweden, where obese women had higher rates of incontinence and lower satisfaction than normal‐weight women 10 years after surgery [2].

Adding to the safety aspect of MUS surgery, we would also highlight the results from a recently published paper from our study population showing that negative impact of urinary incontinence on sexual life decreased after MUS surgery [3]. Persistent pain after MUS in this population was infrequent (3%–4%) with no difference between sexually active and inactive women.

Additionally, we would emphasise the importance of having national quality registries with good coverage and data quality when it comes to surgeries that involve implants, whether they are synthetic, autologous or xenografts. Good registries are particularly useful when new techniques are implemented as they can discover serious adverse events early. Events may occur infrequently at the individual hospital or for the individual surgeon but show a systematic pattern when results from all reporting hospitals are continuously monitored and analysed. Our study demonstrates the value of such a high coverage quality register. In the Norwegian Female Incontinence Registry, preoperative and surgical data as well as results from a mandatory 6–12 months follow‐up are continuously reported. This registry prepares several annual reports to reporting hospitals on results and complications in which results from each hospital department are compared to a national average [4]. We suggest that countries where polypropylene MUS have been banned might benefit from having quality registries in place before reintroducing MUS. Another important factor adding to safety is that everyone who performs MUS surgery in Norway has received training from an experienced surgeon in a one‐to‐one setting for at least 10 surgeries before performing them on their own, which is considered good clinical practice.

Summing up, we are pleased that our study contributes to the robust evidence supporting the safety and efficacy of MUS surgery. We share the commitment to precise risk assessment and patient‐specific evaluations, and we think standardised preoperative assessment and quality registries are important to enable women and surgeons to make safe, evidence‐based and informed decisions.

Thank you again for your valuable comment and for highlighting the significance of our findings.

Sincerely,

Dr. Berit Rein Solhaug, Dr. Rune Svenningsen, Dr. Maria Øyasæter Nyhus and Dr. Ingrid Volløyhaug

## Author Contributions

B.R.S.: writing the letter. R.S., M.Ø.N. and I.V.: editing the letter.

## Conflicts of Interest

The authors declare no conflicts of interest.

## Data Availability

Due to legal issues supporting data is not available. The participants of the study did not give written consent for their data to be shared publicly.
